# AutoSOME: a clustering method for identifying gene expression modules without prior knowledge of cluster number

**DOI:** 10.1186/1471-2105-11-117

**Published:** 2010-03-04

**Authors:** Aaron M Newman, James B Cooper

**Affiliations:** 1 Biomolecular Science and Engineering Program, University of California, Santa Barbara, CA 93106, USA; 2 Molecular, Cellular, and Developmental Biology, University of California, Santa Barbara, CA 93106, USA

## Abstract

**Background:**

Clustering the information content of large high-dimensional gene expression datasets has widespread application in "omics" biology. Unfortunately, the underlying structure of these natural datasets is often fuzzy, and the computational identification of data clusters generally requires knowledge about cluster number and geometry.

**Results:**

We integrated strategies from machine learning, cartography, and graph theory into a new informatics method for automatically clustering self-organizing map ensembles of high-dimensional data. Our new method, called AutoSOME, readily identifies discrete and fuzzy data clusters without prior knowledge of cluster number or structure in diverse datasets including whole genome microarray data. Visualization of AutoSOME output using network diagrams and differential heat maps reveals unexpected variation among well-characterized cancer cell lines. Co-expression analysis of data from human embryonic and induced pluripotent stem cells using AutoSOME identifies >3400 up-regulated genes associated with pluripotency, and indicates that a recently identified protein-protein interaction network characterizing pluripotency was underestimated by a factor of four.

**Conclusions:**

By effectively extracting important information from high-dimensional microarray data without prior knowledge or the need for data filtration, AutoSOME can yield systems-level insights from whole genome microarray expression studies. Due to its generality, this new method should also have practical utility for a variety of data-intensive applications, including the results of deep sequencing experiments. AutoSOME is available for download at http://jimcooperlab.mcdb.ucsb.edu/autosome.

## Background

High-throughput whole-genome expression data generated by microarray and deep sequencing experiments hold great promise for unraveling the genetic logic underlying diverse cellular events and disease. Without the application of sophisticated bioinformatics and statistical methods, however, these enormous datasets invariably defy human analysis. For example, microarray experiments generally yield tables of expression data in which rows represent 20,000 to 50,000 different gene probes, and columns (usually 4-20) generally represent a wide variety of different cellular phenotypes. Such massive, high-dimensional datasets are increasingly generated by 21^st ^century research technology, and robust and practical methods for finding natural clusters in complex microarray data will have broad application beyond bioinformatics in in data-intensive fields ranging from astrophysics to behavioral economics.

Several methods have come to predominate the clustering of microarray data, none of which is ideally suited for identifying the complex systems-level interactions in genome biology [1-3]. A common approach uses bottom-up hierarchical clustering (HC) to build a dendrogram representing a series of clusters and sub-clusters, with cluster number ranging between one (all the data in one cluster) and the dataset size *N *(each data point in its own cluster). A discrete partitioning in HC requires "pruning" the tree into a known number of clusters. Methods for predicting the number of clusters in a dendrogram vary in predictive accuracy and efficiency [3,4]. Also, since HC greedily merges all of the data points into a locally connected dendrogram, local decisions about cluster membership can misrepresent global cluster topology [5]. Another strategy uses K-means clustering to produce a clean partitioning of a large dataset by minimizing the statistical variance within *k *clusters of *d *dimensions. The number of clusters, *k*, is the key parameter for K-means partitioning, and a cluster number prediction algorithm is also important for accurately selecting *k *without prior knowledge [3,4]. K-means clusters are generally limited to hyper-spherical geometries, and the requirement that all data must belong to some cluster may poorly represent relationships in a dataset containing outlier data points.

Over the past decade, many additional unsupervised clustering strategies have been proposed [6,7]. For example, Affinity Propagation uses an instance of the max-sum algorithm to identify exemplar data points that represent cluster centers in the dataset, but is generally restricted to symmetrical clusters, and requires a 'preferences' parameter that ultimately determines the number of clusters [8]. A different approach, non-Negative Matrix Factorization (nNMF), constitutes a class of matrix multiplication techniques that has shown utility for identifying compact, well-defined clusters in noisy datasets [9]. Like K-means and HC, nNMF requires an external cluster number prediction method (e.g. cophenetic correlation) and manual analysis to select the final partitioning. Spectral Clustering methods utilize linear algebra to perform an eigenvector decomposition of input data followed by application of a suitable clustering method (often K-means) to cluster the transformed data points. Although spectral clustering methods have a mathematically robust foundation and work well for identifying clusters of diverse shapes, eigenvector decomposition steps are computationally-intensive, and spectral clustering also requires cluster number as input [10]. Unless data points are represented sparsely, Spectral Clustering and Affinity Propagation both require O(*N^2^*) space for *N *data points resulting in poor scalability for very large datasets such as whole genome expression data. Finally, most modern methods are not sensitive to outlier data points, a potentially critical limitation for cluster analysis of noisy gene expression datasets [7].

A powerful machine learning method widely used for the visualization of high-dimensional data, called the Self-Organizing Map (SOM), also has applications in data clustering [11-17]. To identify *k *clusters, SOM algorithms randomly initialize a regular lattice of *k *nodes, and then through an iterative learning process, similar input data points move toward each other in the lattice and dissimilar input data points move away from each other. As commonly applied, SOM clustering requires *a priori *knowledge of cluster number and only finds clusters with hyper-spherical geometries. A useful feature of the trained SOM is the U-Matrix, which provides a quantitative description of discontinuity in the map. By liberally allocating nodes in the lattice, U-Matrices can be used to identify potential cluster borders [13]. Two recent methods that exploit U-Matrices for clustering include virtual flooding of U-Matrix "valleys" to create cluster "islands" [14], and HC of the U-Matrix using novel cluster merging criteria [15]. These approaches, however, are highly sensitive to critical SOM parameters such as grid node number [15], or grid topology and cluster shape [14]. In addition to novel variants of the SOM that achieve explicit clustering of the node lattice, traditional clustering algorithms, like K-means, have also been applied to the SOM node lattice, though the number of clusters present in the trained map still requires external prediction and may not be accurately identified with circular cluster geometries [16,17]. In addition, the stochastic initialization of the node lattice required for proper self-organization leads to significant output variation for SOM-based clustering strategies. Taken together, these problems have limited the utility of SOM approaches for unsupervised clustering of microarray data.

Analysis of data generated by high-throughput biology experiments would greatly benefit from a facile unsupervised clustering method that addresses the drawbacks of traditional and modern clustering methods (i.e. need for cluster number prediction, restricted cluster geometry, lack of outlier detection, output variance, and poor scalability to large datasets). Here we report a novel SOM-based method for Automatic clustering using density-equalized SOM Ensembles, or AutoSOME. This new method leverages the proven strengths of the SOM for dimensional reduction and spatial organization of large high-dimensional datasets, while addressing major limitations of general data clustering strategies. After using an SOM for initial data organization, AutoSOME applies a density equalization technique from cartography [18] to rescale the SOM output lattice, utilizes a minimum spanning tree approach from graph theory to identify data clusters and outliers, and then employs an ensemble resampling technique over multiple SOM runs to stabilize the output [19]. The performance of AutoSOME is evaluated using several benchmark datasets, including standard machine learning datasets and publicly available cancer and stem cell microarray data. Our results demonstrate that AutoSOME benchmarks favorably against other clustering methods with the significant advantage that AutoSOME is able to identify the number of clusters in the input dataset given an intuitive p-value threshold. In addition, when applied to transcriptome analysis, AutoSOME readily identifies global variation in tumor cell gene expression that is missed by other methods [5,9]. A network visualization of the AutoSOME output powerfully illustrates the underlying fuzziness found in these cancer cell transcriptome data. Finally, the utility of the new method for gene co-expression analysis is demonstrated by the use of AutoSOME to identify a module of ~3400 up-regulated genes in human pluripotent stem cells, including 1165 genes constituting a large protein-protein interaction network related to pluripotency. These results establish that AutoSOME is a practical and robust new method for clustering the information content of inherently noisy, often high-dimensional, gene expression data, and for visualizing global transcriptome profiles.

## Results

### Algorithm

**Figure 1:**
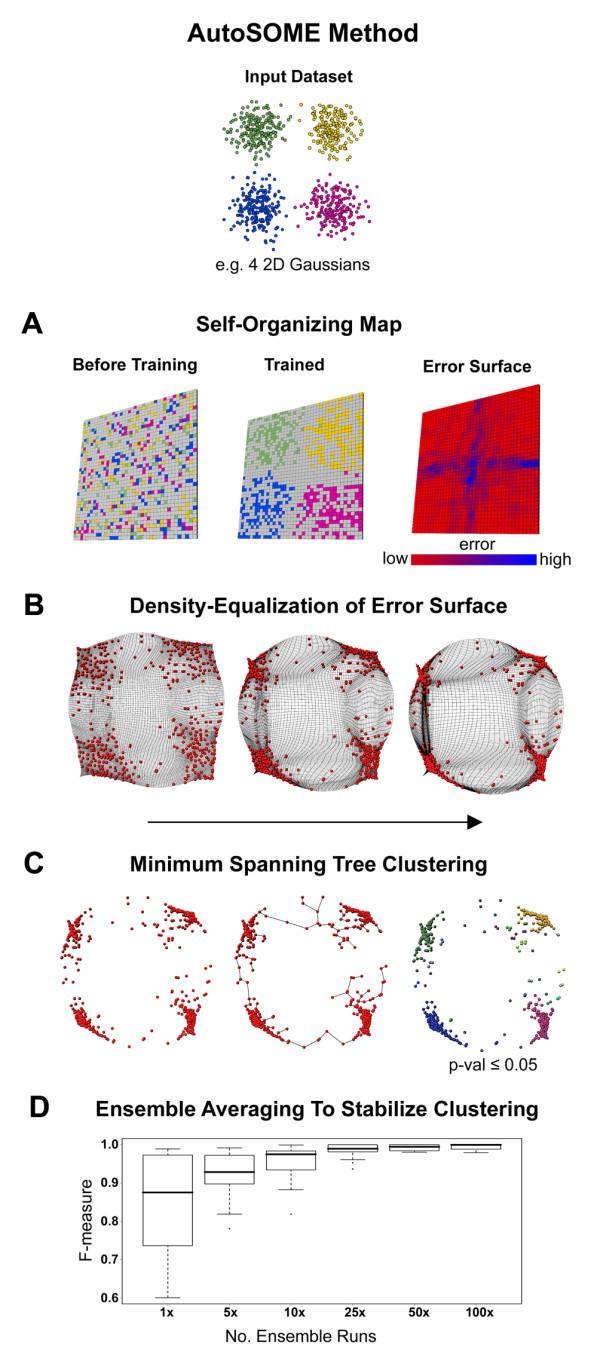
**Overview of the AutoSOME method**. A simple dataset consisting of four 2-D Gaussian distributions of data points, shown on top, is used to illustrate the major steps of the AutoSOME method. (A) Gaussian data points are mapped randomly to the untrained SOM node lattice (left panel), and are organized onto the planar SOM surface after training (middle panel), and the error surface is then computed (right panel), with red representing nodes with highly similar data content compared to neighbors (low error), and blue representing nodes with dissimilar neighbors (high error). (B) A density-equalization procedure treats nodes with high error (Gaussian cluster boundaries) as high density and forces these nodes away from each other while nodes with low error (within clusters) have low density and are forced to aggregate. (C) A Minimum Spanning Tree is built from the rescaled node coordinates, and statistically significant point aggregations of diverse geometries are detected in the dataset using Monte Carlo sampling, resulting, in this case, in the identification of four major clusters corresponding to the four Gaussian point distributions, along with several outlier clusters and singletons (shown by colored nodes in the rightmost image). (D) Impact of number of ensemble iterations on the F-measure, reflecting cluster quality, using the dataset of two interlocking rings (see Figure 2).

The AutoSOME strategy is summarized in Figure 1. A Kohonen SOM is employed to achieve both a dimensional reduction and an initial organization of the input data that preserves local, but not necessarily global, topology (see two leftmost images of Figure 1A) [11,20]. By measuring the similarity between adjacent nodes, an error or dissimilarity surface (i.e. U-Matrix) is calculated, which is cubically rescaled to accentuate separation of individual data clusters. Using the error surface, an aggregation of similar data in the SOM is achieved by repositioning the SOM nodes using the density-equalizing cartogram algorithm from geography (Figure 1B) [18]. In this case, the density-equalizing algorithm treats the high error, or high discontinuity, regions in the map as high density, and the low error regions as low density, and uses a diffusion-based approach to globally equalize the density of the error surface across the entire map. Discrete data clusters of diverse geometries are then identified from the density-equalized SOM using the minimum spanning tree from graph theory, as shown in Figure 1C[21]. Only minimum spanning tree edges that meet a specified p-value threshold are kept, resulting in the identification of statistically significant node clusters and outliers.

**Figure 2:**
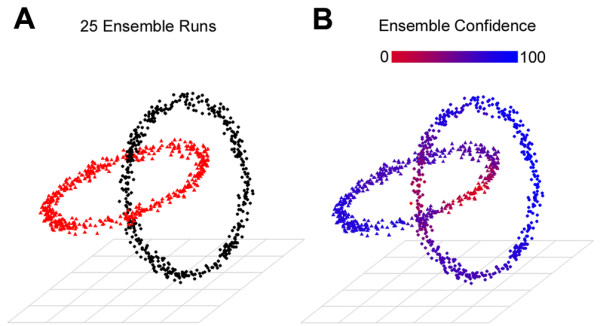
**Clustering of the interlocking rings dataset by AutoSOME with a statistical confidence metric**. The classic and challenging dataset consisting of two orthogonal interlocking rings [13] was analyzed using AutoSOME. (A) Clustering of the rings dataset with increasing ensemble runs, where different colors represent output clusters. Complete resolution of the two rings was achieved following 25 ensemble iterations. (B) A cluster confidence metric projected onto the rings dataset after 25 ensemble runs. The confidence metric for cluster membership is based on the fuzzy clustering ensemble produced by AutoSOME, with conf(*x*, *j*) = 100 (blue) representing the case where data point *x *is always in cluster *j*, and conf(*x*, *j*) = 0 (red) representing the case where data point *x *is never placed in cluster *j*. See Ensemble averaging in Methods for details of the AutoSOME cluster confidence procedure.

A critical issue inherent in all stochastically initialized clustering methods is output variation. To mitigate output variance generated by the SOM step, the AutoSOME method uses an ensemble strategy, merging multiple iterations of the clustering scheme to establish fuzzy clusters that are ultimately resolved by sending data points to clusters where they occur most frequently. This is illustrated using a simple yet challenging benchmark dataset consisting of two clusters of 3- dimensional data that form a pair of interlocking rings. The cluster output stabilized at maximum cluster quality with increasing ensemble runs (Figure 1D), and the intersecting rings data were clearly resolved within 25 iterations (see Figure 2A). As a consequence of the random initialization of each SOM, AutoSOME also provides a statistical confidence metric for membership of every data point to its assigned cluster that is a useful filter for improving the signal to noise ratio (see Figure 2B). All of the important aspects of AutoSOME are detailed in the Methods section, and the software is freely available for download (http://jimcooperlab.mcdb.ucsb.edu/autosome, [22]).

### Testing

To evaluate the unsupervised clustering capability of the AutoSOME method, we compared the performance of AutoSOME with a variety of traditional and modern clustering methods using several benchmark datasets. We also tested AutoSOME on microarray datasets, and compared the AutoSOME output to published results obtained using HC, K-means, and nNMF clustering methods. Finally, we evaluated the ability of AutoSOME to identify modules of co-regulated genes from a large microarray dataset with over 15K expression profiles, and performed a detailed computational analysis of the biological significance of the largest detected co-expression module.

**Figure 3:**
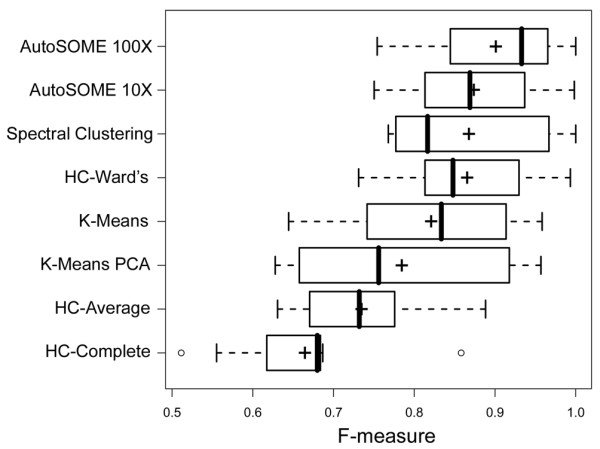
**AutoSOME benchmarking**. AutoSOME performance is compared to common clustering methods by clustering seven diverse benchmark datasets (see Additional file 1 for datasets and details of the entire benchmarking comparison; AutoSOME method with circular node topology is shown here). All clustering methods, apart from AutoSOME, were provided with the benchmark number of clusters for each dataset. Boxplots (created in R [56] using default boxplot parameters) represent the distribution of F-measure scores for all benchmark datasets. An F-measure score of 1 represents a perfect solution. ('+' denotes mean F-measure, and vertical bars denote median F-measure; HC = Hierarchical Clustering; PCA = Principal Components Analysis).

Our benchmarking studies included seven diverse datasets having defined cluster number and geometry, including six datasets widely used by the machine learning community (see Additional file 1, Table S1). We compared our new method to several clustering strategies including K-means, HC, and Spectral Clustering. Since AutoSOME, like all SOM-based methods, performs a dimensional-reduction of input data, we also combined K-means and HC with different dimensional reduction methods for these benchmarking studies (including Principal Components Analysis (PCA), SOM and density-equalized SOM). The correct numbers of clusters in each benchmark dataset were provided for Spectral Clustering and all K-means and HC methods. The accuracy of cluster assignments by each method was assessed with the commonly used F-measure as well as Normalized Mutual Information (NMI) metrics [4,14]. Over a diverse range of benchmark datasets, AutoSOME clustering, with no *a priori *knowledge about cluster number, outperformed most HC methods and performed at least as well as K-means, Ward's HC, and Spectral Clustering methods (Figure 3 and Additional files 1 and 2). We note that K-means and HC have previously been applied to the SOM for clustering [15,16]. Importantly, density-equalization of the SOM node lattice led to a considerable improvement in cluster quality for all tested clustering methods, including K-means, compared to clustering the unscaled lattice (Additional files 1 and 2).

**Figure 4:**
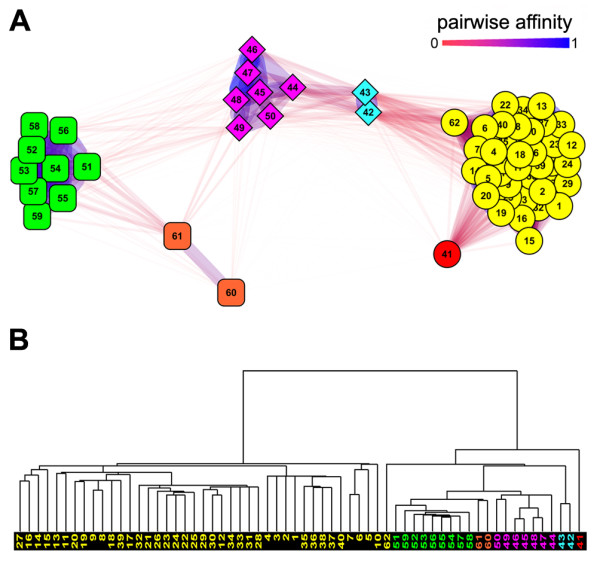
**Fuzzy cluster network highlights variation among tumor cell lines**. (A) Network diagram illustrating the AutoSOME transcriptome analysis of 42 Diffuse Large B-cell Lymphoma (DLBCL) cell lines (circular nodes), 9 Follicular Lymphoma (FL) cell lines (diamond nodes), and 11 Chronic Lymphocytic Leukemia (CLL) cell lines (rounded-square nodes) [5,23]. Nodes are colored by cluster membership. Numbers represent individual cell lines according to their order on the original microarrays. Edges represent the *pairwise affinity *between any two cell lines, defined as the extent to which particular pairs of cell lines are co-clustered by AutoSOME (0 = cells are never co-clustered to 1 = cells are always co-clustered). The diagram was generated in Cytoscape 2.6.0 using the Edge-weighted Spring Embedded layout algorithm [34]. (B) HC of the same cell lines using Uncentered Correlation and Average-Linkage. Cell lines are numbered and colored as in Panel A. The cancer dataset was hierarchically clustered using the software tool, Cluster [1] and the resulting dendrogram was visualized using Java TreeView [57].

Whole-genome expression data are commonly represented in tabular form, where columns are feature vectors reflecting individual cellular transcriptomes and rows are feature vectors representing the expression of individual genes. Clustering methods applied to these data identify distinct cellular transcriptome classes or modules of co-expressed genes or gene variants. We tested the ability of AutoSOME to cluster transcriptome data using a previously "filtered" cancer cell expression dataset representing 2093 gene probes, and three lymphoma tumor types (42 Diffuse Large B-cell Lymphoma (DLBCL) lines, 9 Follicular Lymphoma (FL) lines, and 11 Chronic Lymphocytic Leukemia (CLL) lines) [5,23]. AutoSOME output is effectively visualized using a network diagram in which nodes represent the transcriptome of each tumor line and edges between nodes are weighted by the fraction of times specific cell pairs were co-clustered by AutoSOME over all ensemble iterations. As shown in Figure 4A, AutoSOME effectively partitions 57 of 62 cell lines into three major clusters. The remaining five lines were identified as outliers and clustered into three classes, two FL, two CLL, and a singleton DLBCL. In all cases these outlier transcriptomes are most closely related to the large cluster representing the correct tumor types. None of the cell lines were misclassified by AutoSOME. By contrast, as previously shown, when provided the "correct" number of tumor classes, *k *= 3, K-means forces every transcriptome into one of the three clusters, and makes one misclassification [5]. We used HC to construct a dendrogram representing this data (Figure 4B), but simple orthogonal slices of this tree are unable to cleanly resolve the three tumor classes (see [5]). Three major trunks on the hierarchical tree, involving 56 of 62 branches, can be manually identified, while the remaining six branches include all five outlier lines found by AutoSOME and an additional DLBCL singleton. Because local distance decisions are used to build hierarchical trees, relationships of outlier lines to the major clusters can be lost. This limitation is illustrated in the dendrogram shown in Figure 4B where both singleton DLBCL lines cluster closer to the FL/CLL lineage than the DLBCL lines. In contrast, AutoSOME effectively captures the relationships between outlier transcriptomes and the three major tumor types.

**Figure 5:**
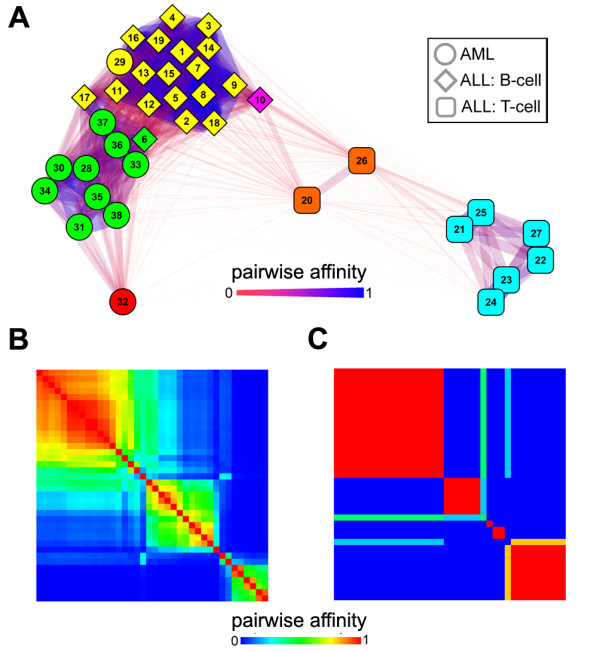
**Robust resolution of fuzzy clusters**. (A) Fuzzy cluster network showing the AutoSOME transcriptome analysis of 11 Acute Myelogenous Leukemia cell lines (AML, circles) and 27 Acute Lymphoblastic Leukemia cell lines (ALL), comprised of 19 B-cell lines (ALL-B, diamonds) and 8 T-cell lines (ALL-T, rounded-squares) [9,24]. Nodes are colored by cluster membership, and numbers represent individual cell lines as originally ordered [9]. Cytoscape network parameters are the same as those used in Figure 4. (B) The same AutoSOME output is shown by a hierarchically reordered heat map, illustrating the considerable fuzziness of cell-cell co-clustering over 500 ensemble iterations (*E *= 500). The diagonal represents self-comparison of each cell line while off-diagonal colors denote cell-cell pairwise affinities as defined in Figure 4. (C) A hierarchically reordered heat map of final cluster assignments averaged over five independent AutoSOME runs (each *E *= 500) illustrates the consistent resolution of the fuzzy clusters into discrete, non-overlapping clusters. Hierarchically reordered heat maps shown in Panels B and C were generated using PermutMatrix software [58].

A relatively new method, based on non-Negative Matrix Factorization (nNMF), has shown utility for transcriptome clustering given a pre-specified number of clusters manually estimated using a cophenetic correlation procedure [9]. To compare AutoSOME to nNMF clustering, we analyzed the cancer transcriptome dataset used by [9], consisting of 5000 gene probes representing 11 acute myelogenous leukemia (AML) tumor lines and 27 acute lymphoblastic leukemia lines, including 19 B-cell (ALL-B) and 8 T-cell (ALL-T) tumor lines [24]. A network visualization of the AutoSOME output (Figure 5A) shows that AutoSOME places 34 of the 38 lines into three major clusters, and identifies outlier data representing a cluster of two ALL-T lines, and singleton ALL-B and AML lines. Like nNMF, AutoSOME makes two classification errors in clustering these data (lines 6 and 29 according to the original cell sample labels, or lines 6 and 10 in the Supplemental Fig. nine published by Brunet et al. [9]).

An important drawback of previous SOM-based clustering methods, as noted in [9], is the instability of cluster assignments (i.e. output variation), ultimately due to the random initialization of the SOM node lattice. Because AutoSOME merges individual runs with an ensemble averaging approach, the method should, in principle, tackle this limitation for both clean and noisy datasets. To measure output variance, we use pairwise affinity, a co-clustering metric defined as the fraction of times a given pair of cell lines cluster together. Pairwise affinities can range from 0 (cells never co-cluster) to 1 (cells always co-cluster). As expected for an SOM-based method applied to noisy microarray data, AutoSOME output appears meta-stable. This is illustrated in Figure 5B by a heat map representing the pairwise co-clustering of leukemia cell lines over 500 ensemble iterations. By combining the discrete output from five separate AutoSOME runs (each with 500 ensemble iterations), pairwise affinities demonstrate that AutoSOME robustly resolves fuzzy clusters into discrete classes over independent runs (Figure 5C). Rather than being a limitation, AutoSOME leverages the random initialization of the SOM node lattice to sample a larger solution space and effectively capture fuzzy data relationships. In contrast, nNMF like K-means, requires an explicit cluster number *a priori *and overlooks outlier cell lines that represent the natural fuzziness in whole genome expression data. We also note that the random initialization of the node lattice naturally renders AutoSOME insensitive to the order of input data points.

**Figure 6:**
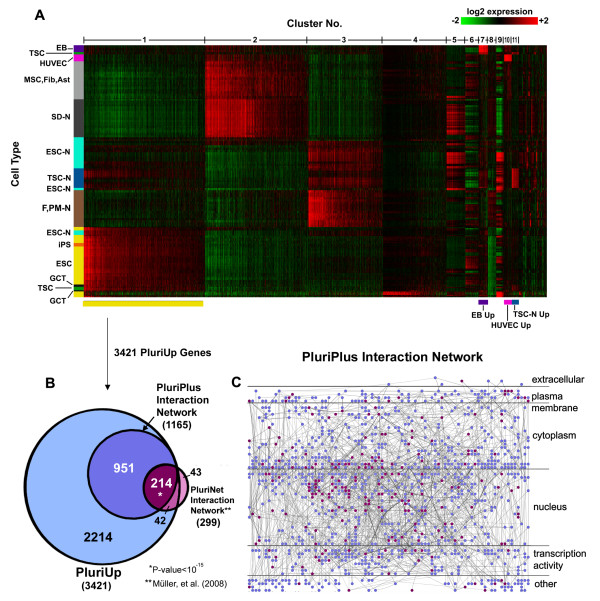
**Genes significantly up-regulated in pluripotent stem cells**. (A) Heat map representation of AutoSOME co-expression analysis of the microarray data GSE11508 [26] showing all gene expression clusters. The heat map is ordered from left to right by decreasing cluster size, and hierarchically ordered from top to bottom by whole-transcriptome expression. EB, embryoid bodies (purple); TSC, teratocarcinoma cells (green); HUVEC, human umbilical vein endothelial cells (pink); [MSC, Fib, Ast], mesenchymal cells, fibroblasts, and astrocytes (light gray); SD-N, surgically-derived neural progenitors (dark gray); ESC-N, embryonic stem cells differentiated into neural stem cells (light blue); TSC-N, teratocarcinoma cells differentiated into dopaminergic neurons (dark blue); [F, PM-N], fetal and post-mortem feeder cells (brown); iPS, undifferentiated induced pluripotent stem cells (orange); ESC, undifferentiated embryonic stem cells (yellow); GCT, germ cell tumor cells (black). Cluster No. 1 represents genes up-regulated primarily in pluripotent stem cells (PluriUp). Several other clusters are labeled below the heat map. Cell lines were hierarchically reordered using Cluster software [1] and the heat map was generated with Java TreeView [57]. (B) Venn diagram demonstrating that the PluriUp gene cluster shares 256 genes with PluriNet, a highly significant overlap as determined by Fisher's Exact Test (p < 10^-15^). (C) The highly interconnected protein-protein interaction network called PluriPlus, consisting of 1165 PluriUp genes, is illustrated as a network diagram. Genes are positioned in the network according to subcellular localization (Gene Ontology from Human Protein Reference Database [31]). Genes found in both PluriUp and PluriNet are colored purple, and genes found exclusively in PluriUp are colored blue. The network diagram was generated using the Cerebral plugin [59] of Cytoscape 2.6.0 [34].

Gene co-expression analysis is used to globally identify sets of genes with similar patterns of expression that underlie important cellular phenotypes. By finding gene modules correlating with differentiation, stress resistance, disease, or pluripotency, for example, co-expression analysis can reveal significant systems-level regulatory networks, and represents another important application of unsupervised clustering. We tested the ability of AutoSOME to identify co-expressed genes by reanalyzing a large, recently published Gene Expression Omnibus [25] dataset (GSE11508) comprised of diverse stem cell and somatic cell phenotypes [26]. Transcriptome clustering of this dataset using a bootstrapped version of nNMF, followed by a comparison of transcriptome classes for significantly enriched interaction networks, was used to identify PluriNet, a protein-protein interaction network consisting of 299 genes significantly associated with pluripotency. AutoSOME co-expression clustering of the GSE11508 data identified 48 distinct gene co-expression clusters, and one singleton gene. Several of the smaller clusters correspond to known phenotypic classes, including umbilical vein endothelial cells, embryoid bodies, and undifferentiated teratocarcinoma cells. The largest cluster contains approximately 3400 genes that are up-regulated in pluripotent stem cells (Figure 6A). Based on this co-expression pattern, this gene module is called PluriUp.

We further analyzed the PluriUp gene set for evidence of biological significance using a variety of approaches. Gene ontology enrichment (Database for Annotation Visualization and Integrated Discovery (DAVID[27]) shows that PluriUp genes are significantly enriched for cell cycle, regulation of transcription/translation, and chromatin remodeling functions, consistent with previous studies [26,28] (see Additional file 3, Table S3). Gene Set Enrichment Analysis (GSEA, [29]) was also used to compare PluriUp genes to other sets of genes reported to be up-regulated in embryonic stem cells (ESCs) [26,30]. As shown in Additional file 3, Table S4, PluriUp genes achieved the greatest enrichment for pluripotent stem cell expression from the GSE11508 dataset. Within the context of six additional GEO datasets consisting of human fibroblast, induced pluripotent stem cell (iPSC), and ESC lines (see Additional file 3, Table S5), the PluriUp genes likewise achieved the highest enrichment scores for both iPSC and ESC lines (see Table S4).

Notably, the PluriUp gene module contains 86% of the PluriNet genes (Figure 6B), a highly significant overlap by Fisher's exact test (p < 10^-15^, Additional file 3, Table S6). Since PluriNet represents only genes whose products compose a large protein-protein interaction network, we examined PluriUp for similar network interactions using a database of 38,806 experimentally verified protein-protein interactions (Human Protein Reference Database (HPRD) release July 6, 2009 [31]). Within PluriUp we identified 1165 genes that encode an interconnected protein network that we call PluriPlus (Figure 6B and 6C; also see Additional file 4, Tables S7-S9 for raw network data and see Additional file 5 for a high-resolution image). Large fractions of both the PluriPlus (200/1165) and PluriUp (459/3421) genes are known to bind at least one of the ESC-specific transcription factors, OCT4, SOX2, and NANOG (p < 10^-5^) [32] (Additional file 3, Table S6). In addition, the PluriPlus interaction network is significantly enriched in genes involved in the Wnt, Notch, EGFR1, and/or TGF-Beta Receptor signaling pathways [31,33] (Table S6). Furthermore, PluriPlus genes show the highest levels of up-regulation in ES and iPS cells, compared to other cellular phenotypes (p < 10^-15 ^by Wilcoxon Rank-Sum Test; Additional file 6, Figure S2). Taken together, these results demonstrate the practical utility of AutoSOME for the identification of robust gene co-expression clusters, such as PluriUp, without prior knowledge of data structure or cluster number.

### Software implementation

The AutoSOME method is implemented as a platform-independent JAVA software tool and is available at [22]. In addition to invocation from the command-line, AutoSOME can be launched within an intuitive Graphical User Interface environment (GUI). The GUI includes several tools for exploring the cluster output, including the generation of publication-quality heat maps as well as real-time editing and filtration of clusters using the confidence metric (see Figure 2B). The AutoSOME webpage also contains all datasets analyzed in this paper, supporting documentation, a tutorial, and instructions to make fuzzy cluster network displays using Cytoscape [34]. For details on how AutoSOME processes input files see *Input *in Additional file 7. In addition to simple tabular input, AutoSOME accepts common microarray file formats: PCL (i.e. Pre-CLuster format implemented in the Cluster software [1]) and raw series matrix text files available from the Gene Expression Omnibus [25].

## Discussion

Increasing advances in computing technologies along with methods for rapidly analyzing diverse living and non-living systems are catalyzing a new era of scientific investigation characterized by a pervasive and critical role for unsupervised data-mining methods. In a recently published comparison of forty clustering algorithms applied to gene expression and network analyses [7], the following set of desirable features for unsupervised clustering algorithms were proposed: 1) scale well in memory and running time with increasingly large datasets, 2) detect distant outliers, 3) produce consistent output regardless of the ordering of input data points, 4) require minimal user input, 5) support both numerical and categorical data types, and 6) identify clusters of diverse geometries. We developed a new unsupervised clustering method for "omics" biology, called AutoSOME, to satisfy the above criteria (with the exception of (5) which remains the subject of future work). We rigorously evaluated the performance of AutoSOME by comparison to other clustering methods including Spectral Clustering and variations of K-means and HC with and without dimensional reduction. Based on two different metrics of cluster quality, AutoSOME, without specification of cluster number, performed at least as well Spectral Clustering, K-means and Ward's HC methods, and achieved better results than three flavors of HC (see Figure 3 and Additional file 1, Figure S1).

AutoSOME has significant advantages over many clustering methods commonly used in bioinformatics research. In particular, AutoSOME identifies clusters of diverse geometries along with outlier singletons without prior knowledge of cluster number, and processes whole genome microarray datasets in practical running time using a desktop computer. AutoSOME is similar in concept to another approach based on moving SOM nodes during the training process to identify aggregated node clusters using hierarchical tree cutting [35]. In contrast to this previous work, however, AutoSOME globally rescales the node lattice after training, identifies clusters using a statistical significance cutoff, and uses ensemble averaging to stabilize results, thus avoiding errors due to local decisions, dendrogram pruning, and stochastic initialization. Another recently developed clustering method based on ensemble averaging of K-means clusters also predicts cluster number and shape, but requires *N*^2 ^space for *N *data points, limiting this method to small datasets [36]. In its current form, the AutoSOME method does not identify genes whose co-regulation is restricted to a subset of arrays (see e.g., [37-39]). This is often accomplished by bi-clustering, a class of techniques that identifies "checkerboard patterns" by the simultaneous clustering of both rows and columns. A bi-clustering extension of AutoSOME is the subject of future work.

Interpretation of whole genome expression data generated by microarray or deep sequencing technologies requires a robust method to compare global gene expression patterns. To illustrate the practical utility of AutoSOME for gene expression studies, we re-analyzed several microarray datasets representing gene expression in tumor cell lines and pluripotent stem cells. Data shown in Figures 4 and 5 demonstrate that AutoSOME identifies important classes of cancer cells. By combining the fuzzy and discrete components of AutoSOME results, significant variation among cancer cells was readily visualized using an intuitive fuzzy cluster network approach. Although the existence of heterogeneity among cancer cells has important implications for cancer biology (e.g., clinical diagnostics, prediction of chemotherapeutic outcomes), such inherent cell variation is not detected by common divisive clustering methods, like K-means, that force all data points, including outliers, into *k *clusters reported as separate and distinct entities. Furthermore, while HC methods, by their nature, show differences within and among clusters, such variation can be difficult to discern in complex dendrograms having numerous branches, although some tree-pruning algorithms are beginning to address this problem [40]. In the cancer cell data originally generated by Alizadeh et al. [23], for example, HC successfully identifies outlier cell lines (Figure 4B), but the three major cell clusters cannot be resolved by horizontally cutting the tree, and the placement of some cell lines on the tree clearly reflects local decisions that distorted the global topology of the data (e.g., tumor lines 62 and 41 in Figure 4). The visualization of fuzzy cluster networks identified by AutoSOME provides a more comprehensive picture of natural cluster structure than common unsupervised clustering methods, and should be valuable as a general strategy to study global cell-cell variation.

Gene co-expression analysis represents another powerful method for elucidating the regulatory logic within genomes, and HC has played a prominent and useful role in unsupervised co-expression clustering [1]. Unfortunately, common HC algorithms scale, at best, quadratically in time with increasing dataset size [3,6]. Thus, to achieve practical computational running times for most HC methods, whole-genome microarray datasets typically need to be reduced in size by filtration of the primary data, often by applying arbitrary differential expression thresholds (e.g. log_2 _fold change between minimum and maximum expression values ≥2). A popular alternative to unsupervised clustering identifies co-regulated genes among a predetermined, usually small, number of sample classes using statistical tests, for example Student's t-test or ANOVA. Like HC, however, these methods also involve filtering genes by arbitrary criteria, such as a statistically significant difference and predetermined minimum fold change (e.g. 2 classes: Student's t-test p < 0.05 and minimum log_2 _fold change = 1.5; e.g. [41]). By filtering the primary data before analysis, both approaches can discard thousands of genes, many of which could have biological relevance (e.g. fold change = 1.4). Further, by averaging across sample replicates to compute fold change between classes, cell samples with stochastic or even meaningful spiking patterns are absorbed and lost, potentially introducing false-positives [42]. By contrast, because AutoSOME efficiently clusters whole-genome datasets without any assumptions about class membership, clustering results are determined by natural cluster structure of the entire dataset. This allows AutoSOME to detect and visualize unexpected expression patterns, such as cell subtypes or stochastic noise. AutoSOME co-expression analysis can also detect biologically significant genes with subtle differential expression patterns that might otherwise be missed. After clustering, appropriate class-based statistical tests like Student's t-test, ANOVA, or Gene Set Enrichment Analysis [29] can be used to evaluate significance.

To demonstrate the capability of AutoSOME for whole-genome co-expression analysis, we reanalyzed a publicly available metadataset (GSE11508) of diverse human cellular phenotypes including 48 ESC and 3 iPSC lines. Application of AutoSOME co-expression analysis to the GSE11508 dataset readily revealed prominent clusters of co-regulated genes, one of which contains >3400 genes primarily associated with pluripotent stem cells. This PluriUp cluster is several times larger than previously reported sets of genes up-regulated in human pluripotent stem cells [30,43], and remarkably, constitutes about 17% of the human genome. A variety of bioinformatics analyses revealed that PluriUp is significantly enriched in ESC-associated cellular functions and genes bound by ESC-associated transcription factors. Due to the relative scarcity of iPS lines in the GSE11508 dataset, we assembled a new metadataset containing 12 fibroblast, 8 ESC, and 42 iPSC lines from multiple reprogramming experiments (see Additional file 3, Table S5), and found that PluriUp is likewise significantly enriched in iPS and ES cell types over fibroblast cell lines, suggesting that PluriUp genes are indeed, pluripotency-associated, and not likely to be an artifact of the primary GSE11508 dataset. Within the PluriUp gene set, we also identified a large protein-protein interaction network significantly up-regulated in pluripotent stem cells containing 1165 genes, or about 6% of the human genome, which substantially expands upon a recently published pluripotent network, PluriNet, containing only 299 genes [26] (see Figure 6 for PluriPlus network, see Additional file 4, Tables S8 and S9, for edges and nodes, respectively; see Additional file 5 for a high-resolution image of the PluriPlus network with HUGO gene names). In addition to sharing 214 genes with PluriNet, PluriPlus is significantly enriched in both important ESC signaling pathways and genes with ESC-associated transcription factor binding sites (see Additional file 3, Table S6). Taken together, both PluriUp and PluriPlus were easily identified, suggesting that a comparable workflow based on AutoSOME co-expression analysis coupled with additional bioinformatics tools can readily lead to the discovery of co-regulated genetic networks from myriad cellular systems.

## Conclusions

We have shown through benchmarking and validation using publicly available machine-learning datasets and microarray data that AutoSOME is a robust cluster discovery method for high-throughput biology. AutoSOME exploits the strengths of the SOM algorithm for unsupervised spatial organization and dimensional reduction of large, unfiltered input datasets while mitigating its shortcomings for data clustering (spatially-fixed lattice of nodes, hyperspherical cluster geometries, output variance) using a novel combination of density-equalization, minimum spanning tree clustering, and ensemble averaging strategies. In addition to predicting the number of clusters without shape restrictions, AutoSOME identifies outlier data points, a potentially critical feature for modeling natural cluster structure that is unavailable in common methods. Further, ensemble averaging reveals the underlying fuzziness of data clusters, which is quantitatively recorded as a cluster confidence metric and usefully visualized by fuzzy cluster networks. Transcriptome analysis using AutoSOME consistently and intuitively characterized significant cell-cell variation in cancer cell lines, and gene co-expression analysis revealed thousands of genes up-regulated in pluripotent stem cells, including 1165 genes composing a large protein-protein interaction network. Based on these results, we conclude that AutoSOME should have immediate utility for researchers seeking to discover natural data classes from a variety of large complex datasets in biology and beyond.

## Methods

### Datasets

Five of the seven benchmark datasets described in Additional file 1, Table S1 (Dermatology (derm), Iris, Breast Cancer Wisconsin (Original) (wisc), Wine, and Zoo), were downloaded from [44]. The other two benchmark datasets in Table S1, synthetic bars consisting of 6 evenly-spaced vertical lines (100 points each) with 1 horizontal line above and another below (152 points each), and interlocking rings ([13], see Figure 2), are available on the AutoSOME website [22]. Columns of all seven datasets were normalized into range 0-100 prior to clustering. The Alizadeh et al. [23] and Goto et al. [24] filtered microarray datasets from different tumor cell lines were downloaded from the authors' website [45] and PNAS [9], respectively. GSE11508 was downloaded from the Gene Expression Omnibus (GEO, [25] as a quantile-normalized file, while the additional GEO datasets [46-50] (listed in Additional file 3, Table S5) were downloaded as raw CEL files and normalized together with Robust Multi-Chip Averaging (RMA) using the Affymetrix Expression Console software.

### Self Organizing Map

Let *T *denote the input dataset consisting of |*T*| vectors with dimensionality *d*. The SOM method utilizes a regular lattice of *n *nodes, which through a process of iterative learning, becomes organized in a manner that preserves and displays local topological relationships among the members of *T*. Our implementation of the SOM consists of a 2-D (circular or square) array of nodes *n*, {*n*_1_, *n*_2_, ..., *n*_*m*_}, where each node *n*_*j *_consists of a feature vector of weights identical in dimensionality to the input *T *[11]. Training of the SOM is accomplished with randomly selected training examples *t *∈ *T *over two phases of *I *iterations each (*I *= 1000 by default), with the second phase devoted to fine-grained learning (learning parameter = 0.9 and 0.1 for first and second phases, respectively; see [11]). Both the learning parameter and neighborhood radius (1/2 grid and 1/4 grid size for first and second phases, respectively) exponentially decrease with increasing iterations. In addition, our SOM implementation automatically computes the number of nodes |*n*| given the input size *|T*| (see *SOM node topology *in Additional file 7 for details).

### Error surface calculation

The error surface *En *represents the continuity among trained nodes and is exploited for clustering by global spatial transformation (see Density equalization of error surface). Note that *En *is analogous in concept to the U-Matrix visualization method [13], and is called 'error surface' due to limitations of the SOM for global topology preservation [20]. Adjacent nodes with high error are very dissimilar while neighboring nodes with low error are likely part of a node cluster (see Figure 1A). *En *is calculated as follows:

, where

i) *Dn*_*j *_= ||*n*_*j*_||,

||·|| = *Euclidean *distance between n_j _and directly adjacent nodes n*

ii) 

iii) Softening parameter *θ *deflates the contribution of higher *Dn*_*j *_to *En*_*j *_when *θ *> 1 (= 1.5 by default), and thus reduces the influence of outlier nodes.

A cubic transformation of the SOM error surface was empirically determined to yield better separation and clustering compared to linear density equalization. The error surface is thus *En*_*j *_← *En*_*j*_^*α*^, ∀*n*_*j*_∈*n*, where α = 3 by default. While more sophisticated methods are possible, such as modeling the error surface by a probability density function [14], the method presented here worked well in our benchmarking experiments.

### Density equalization of error surface

A critical and novel feature of AutoSOME is the application of a Density-Equalizing cartogram (DE) algorithm [18] to *globally *distort the completely trained SOM node lattice such that any clusters present in the node lattice are converted into spatial point aggregations. Input to the DE algorithm is a set of geographic regions and corresponding census values. To meet the input requirements, each node is treated as a unit area square with top-left corner coordinates equal to its SOM grid position. In addition, each error value *En*_*j *_becomes the local population density. Nodes with high error thus have high population density, and will be inflated, while nodes with low error have low density and will be deflated. By distorting each node square in proportion to *En*_*j*_, the density-equalizing spatial transformation, *n*_*j*_^*DE *^← *n*_*j*_, converts implicit SOM cluster structure into explicit spatial point aggregations (see Figure 1B). Note that for efficient implementation, the cartogram dimensions must each be a power of 2 (by default, AutoSOME uses 64 × 64). Final node coordinates are computed as the center of resulting density-equalized boundary coordinates.

### Minimum spanning tree clustering

As illustrated in Figure 1C, to detect clusters *C *of diverse geometries within *n*^*DE*^, all nodes are used to build a Minimum Spanning Tree (MST), *M*. The MST graph connects all nodes *n *by edges *e *with minimum total distance and no loops. The longest edges of *M *are iteratively removed until all edges meet a user-defined p-value threshold (≤ 0.1 by default). The resulting edges e* compose a set of minimum spanning trees M* that define (1+*e*-e*) clusters within the input *T*. Importantly, this process allows for singleton identification. Monte Carlo sampling is used for calculating p-values of all edges in *M *by comparison to edges from β random minimum spanning trees comprised of the same number of nodes in the same bounded space as *M *[21]. By setting β equal to a wide range of values (1-10000), a default value of β = 10 was determined to yield an effective compromise between accurate p-value estimation and practical running time.

### Ensemble averaging

A principal feature of AutoSOME is the implementation of an ensemble resampling method to increase output stability and cluster quality. The algorithm is cyclically run on the input dataset *T *from the SOM through MST clustering steps a total of *E *times. All runs *r*, {*r*_1_, *r*_2_,..., *r*_*E*_}, are then averaged using a novel ensemble procedure based on a previously described method [51] (see *Ensemble averaging *in Additional file 7 for implementation details). In general terms, the number of clusters in each run is initially adjusted to equal the mean number of clusters from all runs *μ*. Next, a matrix **F **is constructed with |*T*| rows and *μ *columns. After integrating all clusters from *r*, **F **will become a fuzzy clustering matrix, whereby each data item can belong to more than one cluster with fractional membership. Finally, **F **is resolved into a discrete set of clusters by placing data points into the clusters where they occur most frequently. Importantly, this process provides a cluster confidence metric for each data point (for details, see *Ensemble averaging, Cluster Confidence Metric *in Additional file 7).

### Microarray data processing and analysis

Both cancer datasets were unit variance normalized and converted into distance matrices using Euclidean distance prior to clustering as described in the *Input *section of Additional file 7. After extensive experimentation using microarray datasets available at [45], Euclidean distance was empirically chosen over Pearson's or Uncentered correlation metrics for this analysis as it gave results closest to previously known cellular phenotypes. For completeness, Euclidean distance and both correlation metrics are included as a user-defined parameter in our implementation of AutoSOME. The GSE11508 dataset was log_2 _scaled followed by unit variance normalization of arrays, median centering of genes and arrays (to eliminate amplitude shifts), and finally, normalization of genes/arrays such that the sum of squares of each row/column = 1. Further, probes of the GSE11508 dataset were converted into Human Genome Organization (HUGO) gene symbols using the IlluminaV1 probe legend obtained from the website that accompanies [26,52]. All gene identifiers were then collapsed into a non-redundant set by averaging expression values for genes represented by more than one probe, resulting in 13,056 genes (*n *for all statistical analyses). Probes without corresponding gene symbols were not analyzed. In addition, updated HUGO symbols (obtained from [53]) were mapped onto the entire PluriUp and PluriPlus gene sets and are made available as Additional file 4, Table S7.

Cancer microarray datasets and the GSE11508 expression dataset were clustered by AutoSOME using 500 and 100 ensemble iterations, respectively. All datasets were run with MST p-value ≤ 0.1. In addition, all microarray datasets were given a maximum SOM grid size of 20 × 20, a minimum of 5 × 5, and run with circular node topology (see *SOM node topology *in Additional file 7 for details). All tests and analyses were performed using a 2.4 GHZ Intel Core 2 Quad CPU with 1.6 GB RAM allocated to the Java Virtual Machine.

### Gene functional analysis

We used a variety of methods to analyze the biological significance of PluriUp and PluriPlus gene sets. Gene Set Enrichment Analysis (GSEA, [29]) takes gene sets, an expression dataset, and phenotype labels as input, and computes enrichment scores of each gene set for one of two phenotypes (e.g. pluripotent stem cells versus other cell types). We used the following gene sets for GSEA: PluriUp gene set (>3400 genes), 532 genes extracted from [30], 299 PluriNet genes [26], and 2000 genes randomly drawn from GSE11508. GSE11508 and the metadataset described in Table S5 were used as the expression datasets for GSEA. DAVID [27,54] was used to compute gene ontological enrichment. To identify genes with ESC-transcription factor binding sites, the PluriUp/PluriPlus gene sets were compared to genes with OCT4, SOX2, or NANOG binding sites reported by [32]. Genes involved in important stem cell signaling pathways were downloaded from [33]. Finally, the PluriPlus interaction network was assembled using the protein-protein interaction dataset downloaded from the Human Protein Reference Database (Release 8, July 6, 2009; [31,55]). To determine statistical significance of overlapping gene sets (e.g. PluriPlus and PluriNet), Fisher's one-sided exact test was used with *n *= 13,056 unique genes (GSE11508 dataset), and was limited to genes contained in the GSE11508 dataset. The Wilcoxon Rank-Sum Test was used to determine the statistical significance of PluriPlus gene expression levels up-regulated in pluripotent stem cells compared to other cell types (see Additional file 6, Figure S2). All statistical analyses were performed using R [56].

## Authors' contributions

AMN conceived of, designed, implemented, and validated AutoSOME, created the AutoSOME webpage, analyzed microarray datasets, and drafted the manuscript. JBC conceived of and validated AutoSOME, analyzed microarray datasets, and drafted the manuscript. Both authors read and approved the final paper.

## Supplementary Material

Additional file 1 **Benchmarking analysis**. Table S1, Description of benchmark datasets; Figure S1, AutoSOME performance compared to seven clustering methods, including six methods with and without four different dimensional reduction techniques; Additional references. Click here for file

Additional file 2 **Table S2**. F-measure and NMI for each benchmarking dataset an clustering method. Click here for file

Additional file 3 **Analysis of PluriUp and PluriPlus biological significance**. Table S3, PluriUp gene ontology functional enrichment; Table S4, Gene Set Enrichment Analysis of PluriUp genes; Table S5, Summary of microarray metadataset assembled from 5 iPSC reprogramming experiments; Table S6, PluriUp and PluriPlus genes significantly overlap with ESC-associated genes. Click here for file

Additional file 4 **PluriUp and PluriPlus gene list and raw interaction network**. Table S7, Updated HUGO gene symbols for PluriUp and PluriPlus; Table S8, Edges of PluriPlus interaction network; Table S9, Nodes and annotation of PluriPlus interaction network. Click here for file

Additional file 5 **High-resolution image of PluriPlus network**. PluriPlus protein-protein interaction network with HUGO gene symbols mapped onto each node (purple nodes = geneshared by PluriPlus and PluriNet [26], blue nodes = gene found in PluriPlus and not in PluriNet). Click here for file

Additional file 6 **Figure S2**. Up-regulation of PluriPlus interaction network in pluripotent stem cells. Click here for file

Additional file 7 **AutoSOME implementation details**. Input, SOM node topology, Ensemble averaging, Additional references Click here for file
