# Supplementary material for: AutoSOME: a clustering method for identifying gene expression modules without prior knowledge of cluster number
Source: BMC Bioinformatics. 2010 Mar 4;11:117. doi: 10.1186/1471-2105-11-117 (PMC2846907; doi:10.1186/1471-2105-11-117)
Supplement: Additional file 1 — Benchmarking analysis. Table S1, Description of benchmark datasets; Figure S1, AutoSOME performance compared to seven clustering methods, including six methods with and without four different dimensional reduction techniques; Additional references. [file 1471-2105-11-117-S1.PDF]

Additional file 1 – Benchmarking analysis
Table S1, Description of benchmark datasets; Figure S1, AutoSOME performance compared to seven clustering methods, including six methods with and without four different dimensional reduction techniques; Additional references

Table S1. Description of benchmark datasets.

Dataset	No. Items	Dimensions	No. Clusters	Items Per Class
bars	904	2	8	100 (6X),152(2X)
derm	366	34	6	112,61,72,49,52,20
iris	150	4	3	50(3X)
rings	997	3	2	499,498
wine	178	13	3	59,71,48
wisc	699	9	2	458,241
zoo	101	16	7	41,20,5,13,4,8,10

Figure S1. AutoSOME performance compared to seven clustering methods, including six methods with and without four different dimensional reduction techniques. A combinatorial array of forty clustering methods was compared with two versions of AutoSOME (square and circular topology). For each method, cluster quality was evaluated using F-measure (A) and Normalized Mutual Information (B) [5, 14]. Six clustering methods: Minimum Spanning Tree (MST), K-Means (K), and Hierarchical clustering with Single-Linkage, Complete-Linkage, and Average-Linkage, and Ward's Method) were combined with four dimensional reduction, or mapping, techniques (Self-Organizing Map (SOM), Density-equalized SOM (DESOM), Principal Components Analysis (PCA), and Sammon Mapping (SM) [S1]). For consistency, dimensionality=2 was used for each mapping method. To measure the impact of dimensional reduction on clustering, all clustering methods, with the exception of MST, were also run on the original datasets without dimensional reduction. We also tested these methods against Spectral Clustering as implemented by default in the kernlab [S2] package of R[56]. K-means and Hierarchical clustering methods were tested using in-house implementations. Cluster number was set to the benchmark standard (see Table S1) for Spectral Clustering, K-Means, and Hierarchical algorithms while the upper p-value threshold was set to 0.1 for MST. All distance matrices for hierarchical methods were computed using Euclidean distance. In addition, circular and square node lattice topologies were compared for both SOM and DESOM (see Additional file 7, SOM node topology). The AutoSOME method is thus abbreviated MST-DESOM-Circle or MST-DESOM-Square. Parameters used were: i) SOM and DESOM, node dimensions scaled within range 30 x 30 to100 x 100 (as described in Additional file 7), ii) SM, 200 iterations, and iii) K, 1000 iterations or convergence. With the exception of Spectral Clustering, the performance of each method with a stochastic component (SOM, DESOM, SM, K) was recorded as the mean F-measure (or NMI) from 20 separate runs with 10 ensemble iterations each. All of these methods performed better with ensemble averaging than without (data not shown). Spectral Clustering results were taken as the mean F-measure (or NMI) from five separate runs. We also include results from running the AutoSOME algorithm once on each dataset with 100 ensemble runs ((100X)_MST-DESOM-Circle and -Square). The final results for each clustering method are shown in boxplot form, summarizing the

performance on all benchmark datasets (vertical bar denotes the median of the cluster quality metric, and '+' denotes mean of the cluster quality metric). All methods are ordered from top to bottom by decreasing mean F-measure (or NMI). All F-measure and NMI values are available as Additional file 2, Table S2.

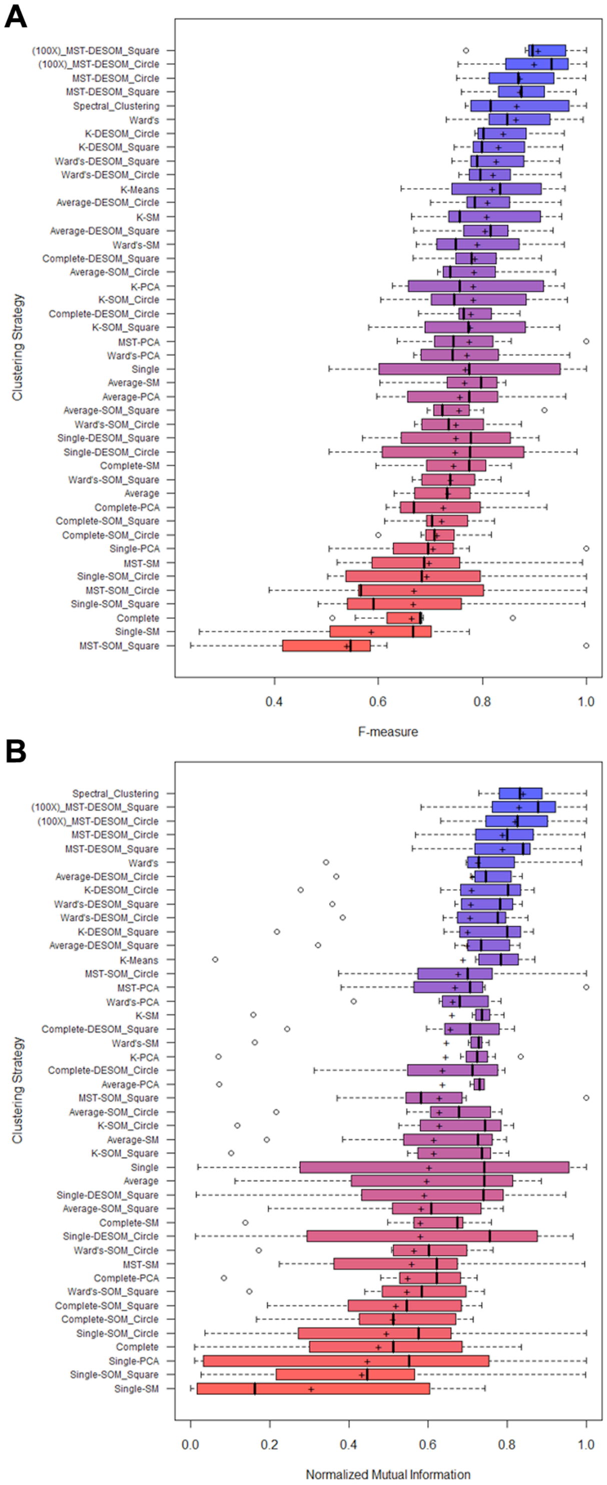

Additional references

S1) Sammon JW: **A Nonlinear Mapping for Data Structure Analysis.** *IEEE Trans. On Computers* 1969, C-18:401-409.

S2) Karatzoglou A, Smola A, Hornik K, Zeileis A: **An S4 Package for Kernel Methods in R.** *Journal of Statistical Software* 2004, **11:**1-20.
