# Supplementary material for: AutoSOME: a clustering method for identifying gene expression modules without prior knowledge of cluster number
Source: BMC Bioinformatics. 2010 Mar 4;11:117. doi: 10.1186/1471-2105-11-117 (PMC2846907; doi:10.1186/1471-2105-11-117)
Supplement: Additional file 3 — Analysis of PluriUp and PluriPlus biological significance. Table S3, PluriUp gene ontology functional enrichment; Table S4, Gene Set Enrichment Analysis of PluriUp genes; Table S5, Summary of microarray metadataset assembled from 5 iPSC reprogramming experiments; Table S6, PluriUp and PluriPlus genes significantly overlap with ESC-associated genes. [file 1471-2105-11-117-S3.PDF]

Additional file 3 – Analysis of PluriUp and PluriPlus biological significance
Table S3, PluriUp gene ontology functional enrichment; Table S4, Gene Set Enrichment Analysis of PluriUp genes; Table S5, Summary of microarray metadataset assembled from 5 iPSC reprogramming experiments; Table S6, PluriUp and PluriPlus genes significantly overlap with ESC-associated genes.

Table S3. PluriUp gene ontology functional enrichment.
All PluriUp genes (see Figure 6 of the accompanying manuscript) were analyzed for functional enrichment using the DAVID (Database of Annotation, Visualization, and Integrated Discovery) bioinformatics resource [27]. Six significantly enriched gene ontology categories are shown. GO=Gene Ontology; BP=Biological Process; MF=Molecular Function.

GO Enrichment (GO Class)	Bonferonni P-value	Benjamini P-value	False Discovery Rate
gene expression (BP)	1.85E-42	2.32E-43	6.75E-43
cell cycle (BP)	1.65E-24	1.27E-25	6.02E-25
methyltransferase activity (MF)	1.60E-07	1.34E-08	9.98E-08
chromatic modification (BP)	5.84E-05	8.99E-07	2.13E-05
mRNA transport (BP)	4.12E-14	1.25E-15	1.50E-14
RNA processing (BP)	1.14E-55	2.86E-56	4.16E-56

Table S4. Gene Set Enrichment Analysis of PluriUp genes.
Gene Set Enrichment Analysis (GSEA, [29]) was used to test the degree of overlap between PluriUp genes and genes enriched in the pluripotency phenotype. A metadataset was assembled from several GEO sources containing iPS, ES, and fibroblast cell lines (20,606 genes; see Table S5). PluriUp achieved the highest enrichment and normalized enrichment scores in both GSE11508 [26] and the metadetaset with a false discovery rate of zero. This result suggests that PluriUp is significantly up-regulated in pluripotency-associated genes. Abbreviations: EScore=Enrichment Score; NEScore=Normalized Enrichment Score; FDR=False Discovery Rate. (In some cases, the number of genes analyzed from each gene set was reduced due to incomplete overlap with the input expression dataset as determined by GSEA.)

Phenotype Tested: Pluripotency (ESC and iPSC)

GSE11508			
Gene Set (No. of genes)	EScore	NEScore	FDR q-value
PluriUp (3421)	0.89	2.25	0
PluriNet (288)	0.77	2.02	0
Brandenberger et al. (403)	0.59	2.01	0.001
Random (1959)	-0.29	-1.37	0.128
iPSC, ESC, Fibroblast Metadataset			
PluriUp (3353)	0.76	1.61	0
PluriNet (285)	0.70	1.53	0.007
Brandenberger et al. (420)	0.67	1.52	0.005
Random (1931)	-0.32	-1.22	0.281

Table S5. Summary of microarray metadataset assembled from 5 iPSC reprogramming experiments. Major attributes of six analyzed microarray datasets representing five human iPSC reprogramming experiments are given [46-50]. All raw microarray CEL files were downloaded from the Gene Expression Omnibus (GEO) at NCBI [25], and normalized by Robust Multi-chip Averaging. Fifteen redundant microarray samples were removed. The BJ1 fibroblast_47 line from GSE9832 was also removed as its whole genome expression signature more closely resembled iPS cells than fibroblasts. The final meta-dataset consisted of 12 fibroblast lines, 8 ESC lines, and 42 iPS lines.

GEO ID	Paper	iPSC Source (Original No. of cell lines)	No. of iPSCs	No. of ESCs
GSE15175	[46]	Foreskin fibroblast (1)	10	5
GSE15176	[46]	Foreskin fibroblast (1)	6	5
GSE13828	[47]	Spinal Muscular Atrophy fibroblasts (2)	3	5
GSE14711	[48]	Parkinson’s disease fibroblasts (1)	8	2
GSE9832	[49]	Neonatal, fetal lung, and ESC-derived fibroblasts (7)	8	1
GSE9709	[50]	Neonatal dermal fibroblast (2)	11	0

Table S6. PluriUp and PluriPlus genes significantly overlap with ESC-associated genes. Overlap of PluriUp, 3421 genes, or PluriPlus, 1165 genes, (see Figure 6 of the accompanying manuscript; both were identified from the GSE11508 dataset) was determined for the following datasets: PluriNet [26], genes with promoters that bind ESC-associated transcription factors (OCT4, SOX2, NANOG) [32], and genes involved in embryonic and developmental signaling pathways [33]. Statistical significance of overlap was computed using one-sided Fisher's Exact Test. The number of genes in each gene set was computed by taking the intersection of each gene set and GSE11508. For example, 2260 genes are reported by Boyer et al. [32] as having promoters that bind ES transcription factors, however, only 1463 of these genes are found in GSE11508.

Dataset (No. Genes)	Overlap (P-value)	
	PluriUp	PluriPlus
Plurinet (299)	256 ($<10^{-15}$)	214 ($<10^{-15}$)
ESC-associated Transcription Factors (1463)	459 (1.6E-6)	200 (5.3E-8)
Signaling Pathway		
Notch (70)	16	13
Wnt (91)	28	22
EGFR1 (154)	28	22
TGFβ Receptor (139)	40	38
Unique Pathway Genes (392)	95 (.83)	79 (2.7E-12)
