# Supplementary figures and images for: AutoSOME: a clustering method for identifying gene expression modules without prior knowledge of cluster number
Source: BMC Bioinformatics. 2010 Mar 4;11:117. doi: 10.1186/1471-2105-11-117 (PMC2846907; doi:10.1186/1471-2105-11-117)

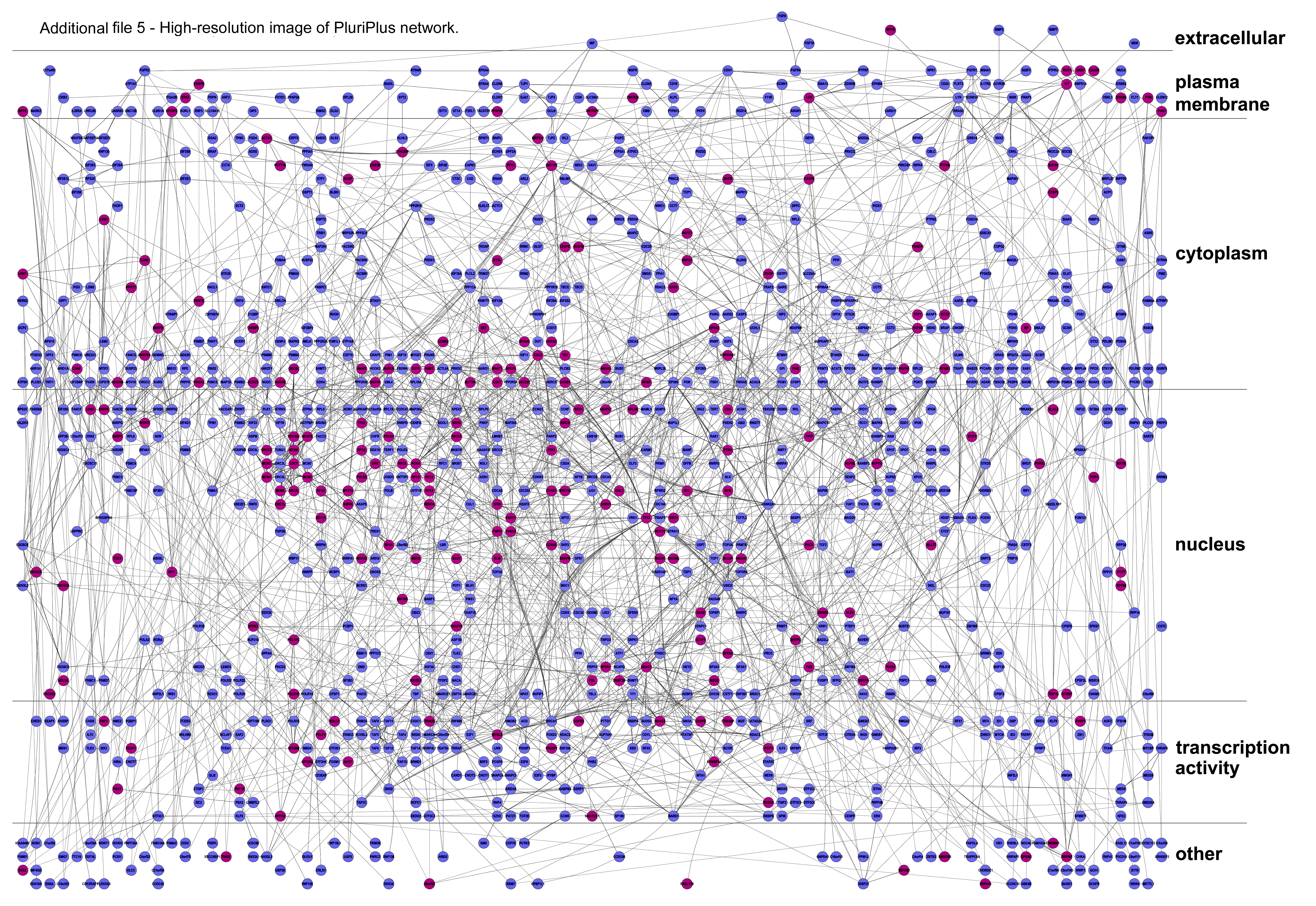

Supplement: Additional file 5 — High-resolution image of PluriPlus network. PluriPlus protein-protein interaction network with HUGO gene symbols mapped onto each node (purple nodes = geneshared by PluriPlus and PluriNet [26], blue nodes = gene found in PluriPlus and not in PluriNet). [file 1471-2105-11-117-S5.JPEG]
