# Supplementary material for: AutoSOME: a clustering method for identifying gene expression modules without prior knowledge of cluster number
Source: BMC Bioinformatics. 2010 Mar 4;11:117. doi: 10.1186/1471-2105-11-117 (PMC2846907; doi:10.1186/1471-2105-11-117)
Supplement: Additional file 6 — Figure S2. Up-regulation of PluriPlus interaction network in pluripotent stem cells. [file 1471-2105-11-117-S6.PDF]

Additional file 6

Figure S2. **Up-regulation of PluriPlus interaction network in pluripotent stem cells.** Expression levels of genes that constitute the PluriPlus interaction network were compared across various cell types. According to the Wilcoxon Rank-Sum Test (calculated in R[56]), PluriPlus is significantly up-regulated in iPS and ES cells over all other tested cell types ($p<10^{-15}$). Cell types are abbreviated as follows: Fib, fibroblasts; HUVEC, human umbilical vein endothelial cells; EB, embryoid bodies; ESC-N, embryonic stem cells differentiated into neural stem cells; TSC-N, teratocarcinoma cells differentiated into neural lineage; TSC, teratocarcinoma cells; iPS, undifferentiated iPS cells; ESC, undifferentiated ES cells. Fib*, ESC* and iPS* represent cells analyzed from the iPS/ES/Fibroblast metadataset (see Additional file 3, Table S5) while all other expression values were obtained from the GSE11508 dataset [26]. For each cell type, expression values were $\log_2$ scaled, averaged across replicates into a single vector of expression values, and to compute fold change, compared to the mean expression vector for all other cell types in the dataset (e.g. GSE11508). When computing up-regulation of iPS or ES cells, both of these pluripotent cell types were removed from the remaining cell types to determine fold change. A Wilcoxon Rank-Sum test was then applied to determine statistical significance of up-regulation for the distribution of expression values of each pluripotent stem cell compared to the other cells. As Fib*, iPS*, and ES* cell lines are not from the GSE11508 dataset, the Wilcoxon Rank-Sum Test was applied to expression distributions of each primary dataset separately. Thus, iPS* and ES* are significantly up-regulated over Fib* while iPS and ES are significantly up-regulated over HUVEC, EB, TSC, TSC-N, and ESC-N. Network heatmaps were visualized using the Cerebral plugin [59] of Cytoscape 2.6.0 [34]. The pyramid of PluriPlus heatmaps is arranged in order of increasing mean expression across all genes in the network going from top to bottom and left to right.

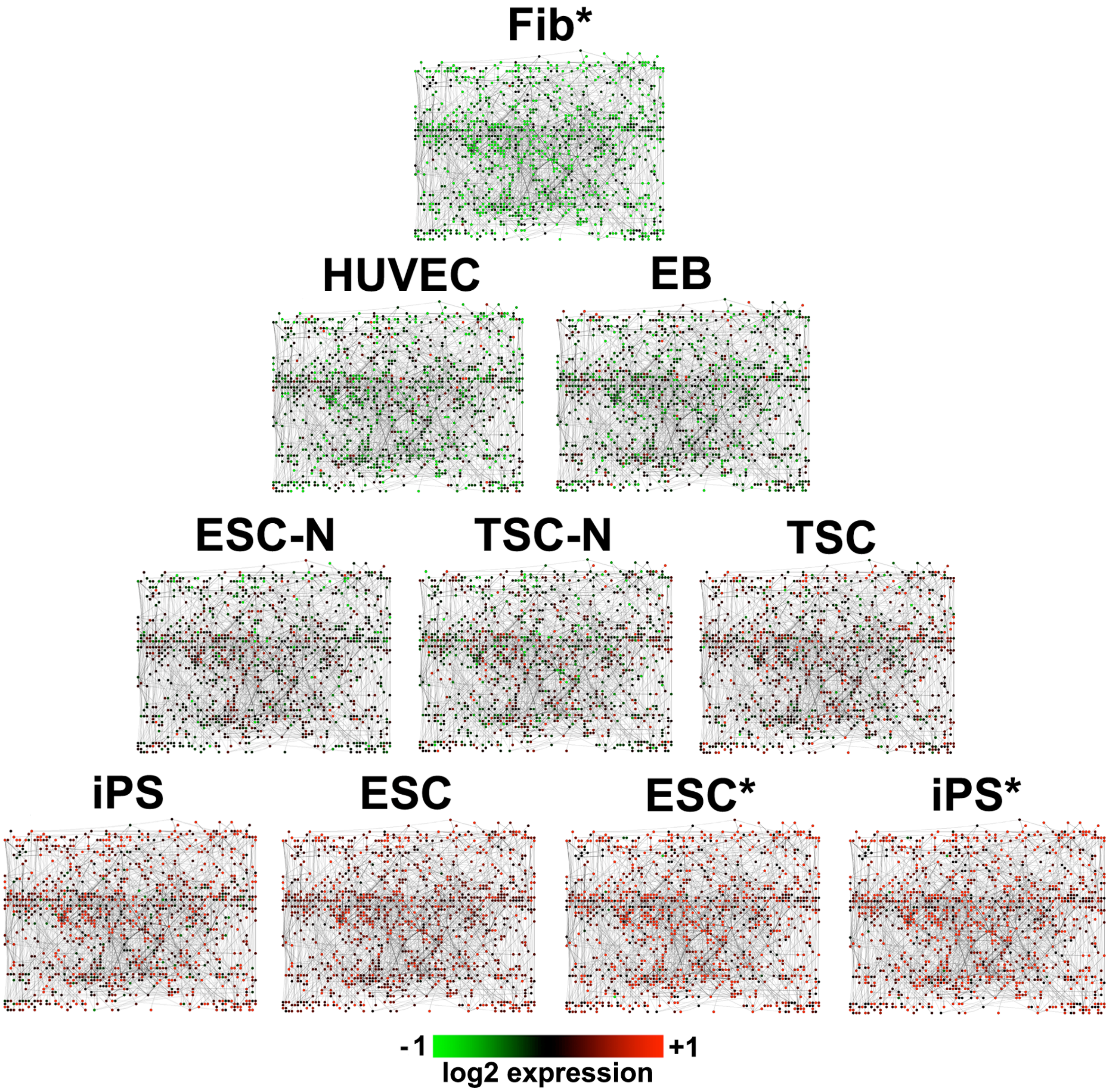
Fib*
HUVEC
EB
ESC-N
TSC-N
TSC
iPS
ESC
ESC*
iPS*
-1
+1
log2 expression
