# Supplementary material for: AutoSOME: a clustering method for identifying gene expression modules without prior knowledge of cluster number
Source: BMC Bioinformatics. 2010 Mar 4;11:117. doi: 10.1186/1471-2105-11-117 (PMC2846907; doi:10.1186/1471-2105-11-117)
Supplement: Additional file 7 — AutoSOME implementation details. Input, SOM node topology, Ensemble averaging, Additional references [file 1471-2105-11-117-S7.PDF]

Additional file 7 – AutoSOME implementation details, Additional references

Input

AutoSOME accepts simple tabular input (tab, space, or comma-delimited) with mandatory row names (column 1) and optional column names (row 1) as well as two commonly used microarray formats: PCL (Pre-CLuster format used in the Cluster software [1]) and Gene Expression Omnibus Series Matrix text files [25]. Input adjustment methods implemented in AutoSOME include $\log_2$ scaling and the following normalization techniques: unit variance or range $[1,x]$ normalization of columns, row/column median centering (subtract median to eliminate amplitude shifts), and row/column sum of squares normalization (squares of all values in each row/column vector sum to 1). In addition, by computing distances between all column vectors in dataset T to create a distance matrix T^D, AutoSOME can detect classes with thousands of variables each, such as distinct cell types. Implemented metrics for construction of T^D include Euclidean distance as well as Pearson and Uncentered Correlation (see [2] for distance metric definitions).

SOM node topology

Two SOM node topologies in 2-D space, circular and square, were implemented. Benchmarking indicates that both topologies yield comparable clustering results (Additional file 1, Figure S1). To liberally allocate the SOM node space, with square topology, the length of one side $l = \sqrt{2|T|}$, while the circular topology is inscribed within the square SOM. The number of nodes automatically scales as a function of $|T|$, the input size:

$$|n| = \begin{cases} \dfrac{\pi |T|}{2} & circular \\ 2|T| & square \end{cases}$$

Restrictions on the node space for large datasets like whole-genome microarrays amount to imposing a maximum number of clusters on the dataset. As most microarray data points (probes) cluster together with minimal variance, limiting the maximum number of clusters to several hundred can still be very effective for identifying significant co-expression signatures. The possibility remains that some other node topology may yield better results for some datasets. For instance, a surface sphere-based SOM would in principle eliminate border effects (see e.g. [63], for description of border effects), thus reducing the number of ensemble iterations needed for convergence. In this case, a surface-sphere density equalization algorithm would also be needed [64].

Ensemble averaging

Notation:

E = No. ensemble runs
T = input dataset
$\mathbf{F}$ = fuzzy cluster matrix
$\mathbf{F}_i$ = fuzzy cluster i, (i>0)
$\mathbf{F}_{ij}$ = fractional membership of data point $T_j \in T$ in $\mathbf{F}_i$
C_k = set of clusters for run k, $(0<k\leq E)$
C_{kp} = the pth cluster of run k, (p>0)

L_p = index of closest fuzzy cluster to cluster C_{kp} (i.e. $L_p \leftarrow p$)

1) Compute μ, the mean number of clusters over all individual AutoSOME runs, r.

2) $\forall r_j \in r$, add or remove edges e^*_j from the set of all edges e_j composing the minimum spanning tree, M_j, such that $(1+e_j-e^*_j) = \mu$

3) Transform cluster set C_1 into matrix **F**, with μ columns and $|T|$ rows, where:

$$\underset{\forall T_j \in T, \forall C_{1i} \in C_1}{\mathbf{F}_{ij}} = \begin{cases} 1 & T_j \in C_{1i} \\ 0 & T_j \notin C_{1i} \end{cases}$$

4) **Set** iterator $k = 2$.

5) **While** $k \leq E$:

For all $C_{kp} \in C_k$ (every cluster p in run k):

i) Find $\mathbf{F}_q$ such that $\mathbf{F}_q = \max_{\forall q \leq \mu} \partial(\mathbf{F}_q, C_{kp})$, where:

$$\partial(\mathbf{F}_q, C_{kp}) = \frac{\sum_{\forall T_j \in C_{kp}} (\mathbf{F}_{qj} + 1)}{|C_{kp}| + \sum_{\forall j \leq |T|} \mathbf{F}_{qj}}$$

Once identified, $\mathbf{F}_q$ is blocked from further merging until k increments.

ii) $L_p = q$ (update index for C_{kp})

End (for)

Update:

i) $$\mathbf{F}_{ij} = \frac{\mathbf{F}_{ij}(k-1)}{k}, \forall i, j \in \mathbf{F}$$

ii) $$\mathbf{F}_{L_p j} = \mathbf{F}_{L_p j} + \frac{1}{k}, \forall T_j \in C_{kp}, \forall C_{kp} \in C_k$$

Increment: $k=k+1$

End (while)

6) Resolve **F** into μ discrete clusters $\{D_1, D_2, \ldots, D_\mu\} \in D$, where:

i) $$D_i \bigcup_{\forall i \leq \mu, j \leq |T|} \delta(i,j)$$

ii) $$\delta(i,j) = \begin{cases} T_j & \mathbf{F}_{ij} = \max_{\forall m \leq \mu}(\mathbf{F}_{mj}) \\ null & \mathbf{F}_{ij} \neq \max_{\forall m \leq \mu}(\mathbf{F}_{mj}) \end{cases}$$

7) **Cluster Confidence Metric:** Due to ensemble averaging, the final set of clusters, D, maps to confidence values (0-100] for each data point. For data point T_j in cluster D_i, confidence is defined as $100^*\mathbf{F}_{ij}$ and is equivalent to the fractional membership of T_j to D_i.

Additional References

S3) Kohenen T: *Self-Organizing Map*. Edited by Huang TS, Kohonen T, Schroeder MR. Springer-Verlag Berlin Heidelberg; 2001.

S4) Gastner MT, Newman MEJ: **Density-equalizing map projections: Diffusion-based algorithm and applications.** *Proc. of the 8th International Conference on Geocomputation* 2005.
